# Sialofucosylation Enables Platelet Binding to Myeloma Cells via P-Selectin and Suppresses NK Cell-Mediated Cytotoxicity

**DOI:** 10.3390/cancers15072154

**Published:** 2023-04-05

**Authors:** Alessandro Natoni, Marina Cerreto, Maria Stefania De Propris, Maria Teresa Petrucci, Francesca Fazio, Stefania Intoppa, Maria Laura Milani, Lucy Kirkham-McCarthy, Robert Henderson, Dawn Swan, Anna Guarini, Michael O’Dwyer, Robin Foà

**Affiliations:** 1 Hematology, Department of Translational and Precision Medicine, Sapienza University, 00161 Rome, Italy; 2 Biomedical Sciences, School of Medicine, National University of Ireland Galway, H91 TK33 Galway, Ireland; 3 Department of Haematology, Galway University Hospital, H71 YR71 Galway, Ireland; 4 Department of Molecular Medicine, Sapienza University, 00161 Rome, Italy

**Keywords:** SLe^a/x^, multiple myeloma, platelets, P-selectin, NK

## Abstract

**Simple Summary:**

Platelet cloaking of tumor cells has been shown to play an important role in tumor metastasis and immune evasion. Multiple myeloma is a tumor of the plasma cells that colonizes different sites of the axial skeleton, including the skull. Herein, we show that specific carbohydrate structures present on the surface of multiple myeloma cells are essential in mediating direct interaction between the platelets and malignant plasma cells, which can be blocked by targeting P-selectin. Platelet binding to myeloma cells inhibits natural killer cell-mediated cytotoxicity, facilitating tumor immune evasion. We propose that platelets are important players in multiple myeloma dissemination, and targeting myeloma–platelet interactions may represent a novel strategy for myeloma treatment.

**Abstract:**

Multiple myeloma (MM) is a plasma cell disorder that develops in the bone marrow (BM) and is characterized by uncontrolled proliferation and the ability to disseminate to different sites of the skeleton. Sialofucosylated structures, particularly Sialyl Lewis a/x (SLe^a/x^), facilitate the homing of MM cells into the BM, leading to resistance to bortezomib in vivo. Platelets have been shown to play an important role in tumor metastasis. Platelets can bind to the surface of cancer cells, forming a “cloak” that protects them from the shear stress of the bloodstream and natural killer (NK) cell-mediated cytotoxicity. In this study, we showed that the presence of SLe^a/x^ induced a strong binding of MM cells to P-selectin, leading to specific and direct interactions with platelets, which could be inhibited by a P-selectin-blocking antibody. Importantly, platelets surrounded SLe^a/x^-enriched MM cells, protecting them from NK cell-mediated cytotoxicity. The interactions between the platelets and MM cells were also detected in BM samples obtained from MM patients. Platelet binding to SLe^a/x^-enriched MM cells was increased in patients with symptomatic disease and at relapse. These data suggest an important role of SLe^a/x^ and platelets in MM disease progression and resistance to therapy.

## 1. Introduction

Selectins constitute a family of three C-type lectins (E-, L- and P-selectin) expressed mostly on the surface of endothelial cells, leukocytes and activated platelets [1]. Like all lectins, selectins bind to glycosylated structures, such as sialylated and fucosylated glycans that decorate proteins and lipids, including the tetrasaccharide Sialyl Lewis a/x (SLe^a/x^). Their major function is to mediate the recruitment of leukocytes to the sites of inflammation and lymphoid tissue [2]. Importantly, all selectins have been shown to contribute to cancer metastasis by different mechanisms [3,4,5]. P-selectin plays a central role in mediating the interactions between cancer cells and platelets [6]. P-selectin is mainly stored within the Weibel–Palade bodies of the endothelial cells or α-granules of the platelets, and it is rapidly translocated to the surface upon activation [7]. Tumor cells can induce platelet activation through different mechanisms leading to the exposure of P-selectin on the surface of platelets, which in turn stimulates their interactions with cancer cells, enhancing tumor metastasis [8,9].

Several studies have demonstrated the important role played by platelets in metastasis [8,10,11,12]. Cancer patients, in particular those presenting with metastatic disease, often experience platelet hyperactivation leading to a high risk of thrombosis, which is the second most common cause of cancer mortality [11,13]. Platelets participate in the metastatic cascade by different mechanisms [6,10]. In particular, it has been shown that activated platelets surround cancer cells, forming a “cloak” that protects malignant cells from the shear force caused by the blood flow [14] and from the immune attack of NK cells [12,15].

A role for platelets has been recently suggested in the progression of multiple myeloma (MM), a malignant plasma cell disorder that develops in the BM [16]. MM is almost invariably preceded by an asymptomatic stage, named monoclonal gammopathy of undetermined significance (MGUS), which eventually progresses to smoldering MM (SMM) and culminates in a symptomatic, full-blown MM [17]. A striking feature of MM is its strong dependence on the BM microenvironment, which stimulates the metastatic spreading of MM cells in different sites of the axial skeleton [18,19]. MM patients often experience platelet hyperactivation even at the MGUS stage, which increases the risk of thrombotic events [16,20]. Indeed, it has been recently shown that MM cells activate platelets, which in turn stimulate MM proliferation in vitro and tumor engraftment in vivo, suggesting that platelets are an important component of the supportive MM microenvironment [16].

We have previously described a subpopulation of MM cells, identified by the monoclonal antibody HECA452, which is enriched for the expression of SLe^a/x^ and E-selectin ligands [21]. The SLe^a/x^-enriched MM population displays an aggressive phenotype characterized by a complete resistance to bortezomib in vivo, which can be reverted by blocking E-selectin with small glycomimetic molecules or by inhibiting sialyltransferases [21,22]. In the present study, we have further characterized the biology of this population of MM cells. We show that SLe^a/x^ induces a strong binding of MM cells to P-selectin, which in turn leads to direct and specific interactions between SLe^a/x^-enriched cells and platelets. Moreover, our results indicate that platelets surround the surface of SLe^a/x^ MM cells and decrease NK cell-mediated cytotoxicity. Finally, we show that MM cells enriched in SLe^a/x^ bind to platelets in BM aspirates, with a higher proportion in patients presenting with symptomatic disease or at relapse. Our study highlights the importance of SLe^a/x^ expression in MM, which endows malignant cells with specific biological traits to allow efficient dissemination and immune evasion during disease progression and treatment.

## 2. Materials and Methods

### 2.1. Cell Lines and Primary Samples

The MM1S and RPMI8226 cell lines were from the American Type Culture Collection (ATCC; Manassas, VA, USA). The SLe^a/x^-enriched cell lines were generated from the parental lines as previously described [21]. Cells were cultured in RPMI-1640 media supplemented with 10% heat-inactivated fetal bovine serum, 50 U/mL penicillin and 50 µg/mL streptomycin, all from Merck Millipore (Rahway, NJ, USA). BM samples from MM patients were obtained with informed consent and ethical approval in accordance with the Declaration of Helsinki and the Ethic Committee of Sapienza University (5816). Patients’ characteristics are summarized in Supplementary Table S1. All reagents were from Merck Millipore unless otherwise specified.

### 2.2. Adhesion Assay

The adhesion assay under static conditions was performed in non-tissue-culture-treated flat-bottom 96-well plates. The wells were coated with 5 µg/mL recombinant human IgG1/E- and P-selectin chimeras (Bio-Techne, Minneapolis, MN, USA) and 5 µg/mL recombinant human IgG1 Fcγ fragment (Jackson Immunoresearch, West Grove, PA, USA) in 100 µL phosphate saline buffer (PBS) overnight at 4 °C. The following day, the wells were washed and blocked with BSA (Bovine Serum Albumin; 1% *w*/*v* PBS) for 1 h at 37 °C. Also, 5 min before the assay, some wells were coated with poly-D-lysine (0.1% *w*/*v* in H_2_O) for 5 min at RT, which served as positive controls. The cells were washed twice in PBS, resuspended at 4 × 10^6^ cells/mL in serum-free RPMI-1640 media supplemented with 5 µM Calcein-AM (PromoCell GmbH, Heidelberg, Germany) and incubated for 15 min at 37 °C in a water bath. After incubation, cells were washed twice in PBS and resuspended in serum-free RPMI-1640 media at 2.5 × 10^6^ cells/mL. Then, 100 µL cell suspension was dispensed into the wells, and the plate was incubated for 2 h at 37 °C. After incubation, the wells were gently washed twice with PBS, and adherent cells were lysed using 100 µL 1% sodium dodecyl sulfate (SDS; Fisher Scientific, Waltham, MA, USA). After a 5 min incubation at RT, fluorescence was measured using the GloMax Discover microplate reader (Promega, Madison, WI, USA) using excitation/emission wavelengths of 475/500–550 nm, respectively.

### 2.3. Rolling Assay

The rolling assay was performed in the 8-channel microfluidic biochips (Cellix Limited, Dublin, Ireland), using a Mirus Evo Nano Pump (Cellix Limited). The biochip channels were coated with 15 µg/mL recombinant human P-selectin (PeProtech, London, UK) in tris-(hydroxymethyl)-aminomethane and hydrochloric acid (Tris·HCl) buffer solution (pH 7.4) supplemented with 1 mM CaCl_2_ and incubated overnight at 4 °C. Each channel was blocked with 1% BSA and incubated at 37 °C for 1 h before the assay. The cells were washed and resuspended in the rolling assay buffer (RPMI-1640 without phenol red supplemented with 1% FBS, 5 mM 4-(2-hydroxyethyl)-1-piperazine ethanesulfonic acid (HEPES) and 1 mM CaCl_2_) at 2 × 10^6^ cells/mL. Then, 8 µL cell suspension was loaded onto the microchannels, and the rolling assay was run at 0.5 dyne/cm^2^ at RT. Cells were monitored in five different positions along the channel using an A-Plan 10X/0.25 objective (Carl Zeiss Microscopy GmbH, Jena, Germany) of an AX10Vert.A1 Microscope (Carl Zeiss Microscopy GmbH). Then, 30 frames per position were collected at 0.5 sec from each other using a 01 QIClick F-M-12 Mono 12-bit camera (QImaging, Surrey, BC, Canada). Images were acquired using the Vena Flux assay software (Cellix Limited), and the analysis was performed using the Image-Pro Premiere software (Media Cybernetics, Rockville, MD, USA). A rolling cell was defined as a cell traveling a distance corresponding to more than its diameter. The number of cells per position was added to obtain the total number of cells per channel, which was then averaged between the numbers of channels.

### 2.4. Flow Cytometry and ImageStream Analysis

Leukocytes from BM samples of MM patients were isolated using an ammonium chloride-based red blood lysis buffer (155 mM ammonium chloride, 10 mM potassium bicarbonate and 0.2 mM ethylenediaminetetraacetic acid [EDTA] tetrasodium salt). Plasma cells were identified using a panel of antibodies, including BV421-CD138 (Clone MI15), BB515-CD38 (Clone HIT2), V500-CD45 (Clone HI30), PerCP-Cy 5.5- CD2 (Clone RPA-2.10) and PerCP-Cy 5.5-CD14 (Clone MφP9), all from BD Biosciences (Franklin Lakes, NJ, USA), PC7-CD19 (Clone J3-119; Beckman Coulter Life Sciences, Lakeview, IN, USA) and PE-CD56 (Clone C5.9; Cytognos, Salamanca, Spain). Malignant plasma cells were defined as CD138-positive, CD38-positive, CD2-negative, CD14-negative and CD19-negative cells. The SLe^a/x^ and CD41/61 were analyzed using the AlexaFluor 647-HECA452 (BD Biosciences) and the APC Vio^®^ 770 CD41/CD61 (Clone REA607; Miltenyi Biotec GmbH) antibodies, respectively. Staining was performed at RT for 30 min in the dark in 200 µL staining buffer (5 mM HEPES, 0.2% BSA [*w*/*v*], 0.09% [*w*/*v*] sodium azide in PBS). After incubation, the cells were washed and resuspended in 500 µL staining buffer. At least 1 × 10^6^ events were acquired using a BD FACS Canto II (BD Biosciences). To monitor CD41/61 in MM cell lines, the initial gating strategy included the BV421-CD138 that allowed the analysis of CD41/61, specifically on MM cells. Having demonstrated that CD41/61 was indeed expressed on MM cells, the CD138 antibody was omitted in follow-up experiments, and MM cells were gated based on forward scatter (FSC) and side scatter (SSC) morphological gate. Staining of MM cell lines was performed on 100 µL staining buffer supplemented with BV421-CD138, PE-HECA452 and APC-CD41/61 antibodies, and incubated for 30 min at RT in the dark. After incubation, the cells were washed and resuspended in 500 µL staining buffer supplemented with 7-aminoactinomycin D (7-AAD, 1:80; Immunological Science, Rome, Italy). At least 1 × 10^4^ events were acquired using a BD FACS Canto II. For the ImageStreaming analysis, the SLe^a/x^ cells incubated with platelets were prepared as described before, resuspended in 50 µL and acquired using an ImageStreamX MarkII imaging cytometer (Merck Millipore), ×60 magnification, with low flow rate/high sensitivity using the INSPIRE software (Merck Millipore).

### 2.5. Platelet Isolation and Incubation with MM Cells

Platelets were isolated from PB samples obtained from healthy volunteers. Undiluted PB was overlaid on a Ficoll density gradient and centrifuged at 200× *g* for 20 min without break. The platelet-enriched fraction was collected and centrifuged at 800× *g* for 10 min without break. Platelets were resuspended in 1 mL serum-free RPMI-1640 and immediately counted using the ADVIA^®^ 560 hematology system (Siemens, Munich, Germany). MM cells were incubated with/without platelets at different ratios in serum-free RPMI-1640 for 30 min at 37 °C in 5% CO_2_. After the incubation, the cells were washed twice with 5 mL serum-free RPMI-1640 and centrifuged at 200× *g* for 10 min without break. Cells were processed for the subsequent analysis.

### 2.6. Analysis of NK Cell-Mediated Cytotoxicity

NK cells were isolated from PB samples obtained from healthy volunteers. Undiluted PB was overlaid on a Ficoll density gradient and centrifuged at 200× *g* for 20 min without break. The platelet-enriched fraction was collected and stored at RT. The mononuclear cells were collected, and NK cells were isolated by negative depletion using the EasySep human NK cell isolation kit and EasySep magnet (Stemcell Technologies, Vancouver, BC, Canada), according to the manufacturer’s instructions. Isolated NK cells were cultured for 7 days in NK MACS medium (Miltenyi Biotech GmbH; Bergisch Gladbach, Germany) supplemented with human serum, IL-2 (500 U/mL,) and IL-15 (140 U/mL), both from Stemcell Technologies. Media was replenished every 3 days. MM cells were incubated with/without platelets and after incubation, cells were cocultured with/without platelet-autologous NK cells at different ratios for 5 h. Cell death was measured by flow cytometry using the Annexin V/7AAD assay (BD Biosciences).

## 3. Results

### 3.1. The Presence of SLe^a/x^ Induces Robust P-Selectin Binding in MM Cell Lines

**Figure 1:**
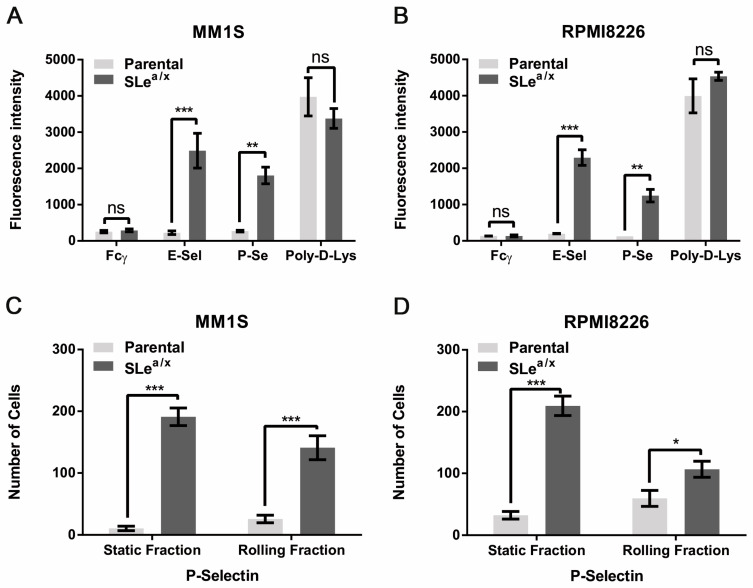
SLe^a/x^-enriched MM cells exhibit robust and specific adhesion and rolling on recombinant P-selectin. (**A**,**B**) Static adhesion assay was performed using Calcein-AM labeled parental and SLe^a/x^-enriched MM1S (**A**) and RPMI8226 (**B**) cells on wells coated with recombinant E- and P-selectin chimera (5 µg/mL). Wells coated with IgG Fcγ (5 µg/mL) and poly-D-lysine (Poly-D-Lys; 0.1% in H2O) were used as negative and positive controls, respectively. Fluorescence intensity was measured at 500–550 nm using a GloMax Discover microplate reader. Bars represent the mean ± standard error of the mean (sem) of three independent experiments performed in triplicate. (**C**,**D**) Parental and SLe^a/x^-enriched MM1S (**C**) and RPMI8226 (**D**) were tested in a rolling assay on recombinant P-selectin chimera (15 µg/mL). Bars represent mean ± sem of three independent experiments performed in duplicate. The two-way ANOVA followed by Sidak’s multiple comparison post-hoc testing was used to determine the statistical significance. *** *p* < 0.001; ** *p* < 01; * *p* < 0.05; ns non-significant.

Since P-selectin has been shown to regulate the interaction between MM cells and the microenvironment [23], we asked whether SLe^a/x^ could modulate binding to P-Selectin. To this end, we performed a static adhesion assay on recombinant P- and E-Selectin using parental as well as SLe^a/x^-enriched MM1S and RPMI8226 cells. Only the SLe^a/x^-enriched MM cells adhered to recombinant E-Selectin, whereas the parental lines did not (Figure 1A,B). Surprisingly, the parental lines did not exhibit adhesion on recombinant P-selectin, while the SLe^a/x^-enriched MM cells displayed robust adhesion (Figure 1A,B), suggesting that the presence of SLe^a/x^ mediated a strong P-selectin binding. To examine whether the SLe^a/x^-enriched MM cells could establish a strong binding to P-selectin also under dynamic conditions, we performed the adhesion and rolling assay under shear stress, which represents a better approximation of the physiologic blood flow in the circulation. Also under dynamic flow, the SLe^a/x^-enriched MM cells displayed a robust adhesion and rolling on recombinant P-selectin compared to the parental lines, which showed a weak adhesion and rolling (Figure 1C,D). The difference in the binding to P-selectin between SLe^a/x^-enriched and parental MM cells was not dependent on the expression levels of P-selectin glycoprotein ligand 1 (PSGL1), the major P-selectin ligand in MM [23], as both cells exhibited similar PSGL1 expression levels (Figure S1A,B). These data strongly suggest that the presence of SLe^a/x^ is essential for proper P-selectin binding.

### 3.2. SLe^a/x^-Enriched MM Cells Interact Directly with Platelets

**Figure 2:**
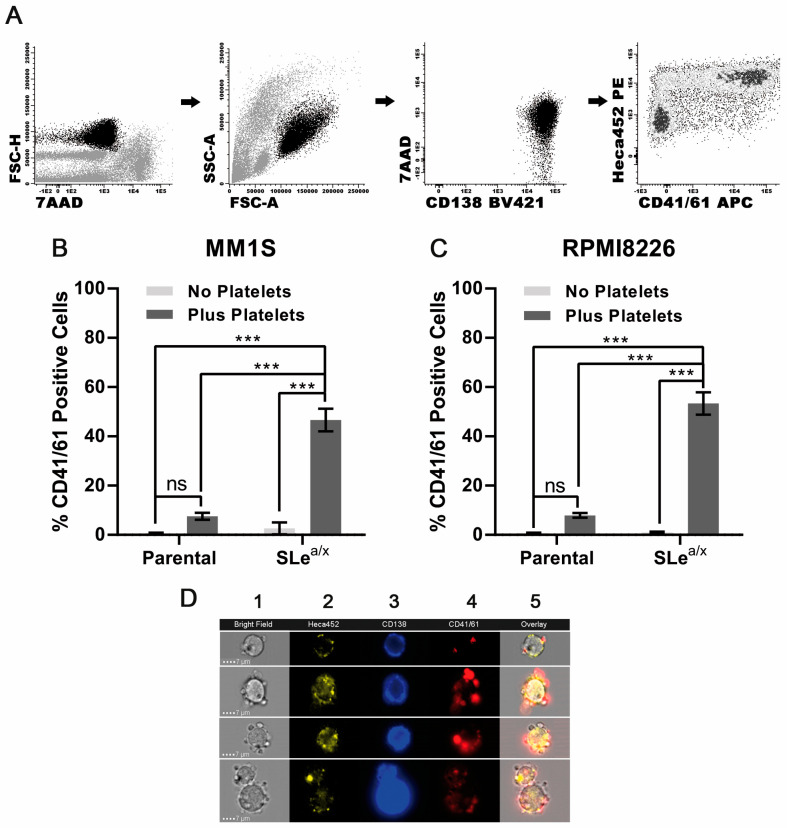
Interaction between MM cells is dependent on the expression of SLe^a/x^. (**A**) The gating strategy was employed to monitor CD41/61-positive cells in CD138-positive MM cells. Dead cells were excluded using the 7-aminoactinomycin D (7AAD). Parental and SLe^a/x^-enriched MM1S (**B**) and RPMI8226 (**C**) cells were co-cultured with/without platelets (platelet:MM ratio 1:100) isolated from the peripheral blood (PB) of healthy donors for 30 min, washed, stained and analyzed by flow cytometry. (**D**) Representative images from the ImageStream analysis of SLe^a/x^-enriched MM1S cells co-cultured with platelets as above. Numbers on the top of the images indicate the following: 1 Bright Field; 2 HECA452; 3 CD138; 4 CD41/41; 5 Overlay. Bar represents 7 µm. Bars represent the mean ± sem of at least three independent experiments. Statistical analysis was performed using two-way ANOVA and Sidak’s multiple comparison test. *** *p* < 0.001; ns non-significant.

It has been shown that P-selectin mediates the interaction between the platelets and different types of cells, including cancer cells [6,7]. Since SLe^a/x^-enriched MM cells displayed a strong binding to recombinant P-selectin, we hypothesized that MM cells could exhibit SLe^a/x^-dependent binding to platelets. We first evaluated platelet binding in the SLe^a/x^-enriched and parental MM1S cell lines. Platelet binding was assessed by flow cytometry using an antibody to CD41/61, also known as GpIIb/IIIa, a surface marker expressed on platelets but absent on MM cells. To determine unambiguously the binding of MM cells to platelets, we used a rigorous gating strategy in which CD41/61-positive cells were analyzed within the MM cells identified by CD138 expression, a marker specific to MM cells (Figure 2A). We also included the HECA452 antibody, which recognizes SLe^a/x^ [24], to examine whether platelets bind specifically to MM cells expressing SLe^a/x^. In the absence of platelets, the SLe^a/x^-enriched and parental MM1S cells did not express CD41/61 (Figure 2B). Upon incubation with platelets, only the SLe^a/x^-enriched but not the parental MM1S cells exhibited strong binding to platelets, as shown by the CD41/61 positivity (Figure 2B). Moreover, almost all CD41/61-positive cells were HECA452 (SLe^a/x^)-positive (Figure S2A), demonstrating the crucial role of SLe^a/x^ in platelet binding. Binding to platelets was also observed in SLe^a/x^-enriched RPMI8226 cells (Figure 2C and Figure S2B). Finally, direct binding of platelets to SLe^a/x^-enriched MM1S cells was confirmed by ImageStream analysis of SLe^a/x^-enriched MM1S cells incubated with platelets, which showed platelets surrounding the HECA452 (SLe^a/x^)-positive MM1S cells (Figure 2D).

### 3.3. Binding of SLe^a/x^-Enriched Cells to Platelets Depends on P-Selectin

**Figure 3:**
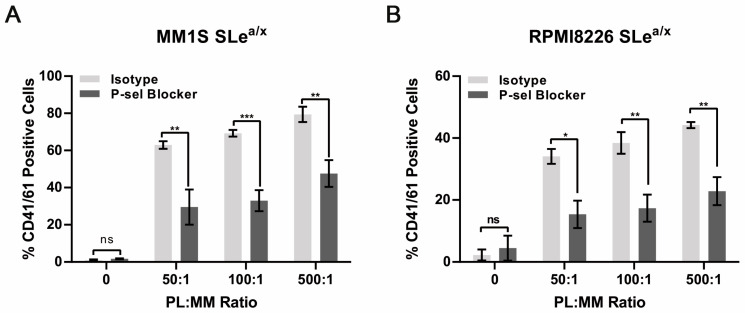
Interactions between SLe^a/x^-enriched MM cells and platelets are inhibited by an anti-P-selectin blocking antibody. (**A**,**B**) SLe^a/x^-enriched MM1S (**A**) and RPMI8226 (**B**) cells were co-cultured with platelets at the indicated platelet:MM ratios for 30 min, washed and then incubated further for 30 min in the presence of an anti-P-selectin blocking antibody or isotype control (both 10 µg/mL). After incubation, the cells were washed and stained by flow cytometry. Bars represent the mean ± sem of at least three independent experiments. Statistical analysis was performed using two-way ANOVA and Sidak’s multiple comparison test. *** *p* < 0.001; ** *p* < 0.01; * *p* < 0.05; ns non-significant.

Next, we examined whether the MM–platelet interactions were P-selectin dependent. SLe^a/x^-enriched MM1S and RPMI8226 cells were co-cultured for 30 min with isolated platelets at different platelet:cell ratios, incubated for 30 min with an anti-P-selectin blocking antibody or isotype control, and then, analyzed by flow cytometry to monitor CD41/61-positive cells. The anti-P-selectin blocking antibody was able to reduce the number of CD41/61-positive cells in both cell lines, indicating that the interactions between MM cells and platelets depended, in part, on P-selectin expressed on platelets (Figure 3A,B). As the P-selectin blocking antibody was added after the cells were incubated with platelets, these results indicate that the MM-platelet interactions could be broken after they were already established, suggesting the displacement of platelets from the surface of the MM cells.

### 3.4. Platelets Partially Protect MM Cells from NK Cell-Mediated Cytotoxicity

**Figure 4:**
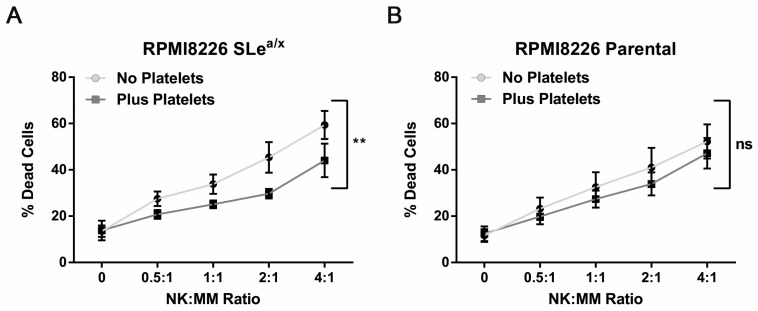
Platelet binding decreases NK-mediated cytotoxicity in sensitive SLe^a/x^-enriched MM cells. SLe^a/x^-enriched (**A**) and parental (**B**) RPMI8226 cells were incubated with/without platelets at a 500:1 platelet:MM ratio as described before. After incubation, cells were washed and incubated for 5 h with platelet-autologous NK cells that have been expanded for 7 days in vitro. NK cells were pre-labeled with the cell trace Carboxyfluorescein succinimidyl ester (CFSE) to distinguish them from MM cells. After 5 h, cell death was assessed by flow cytometry using the Annexin V/7AAD assay. Points on the curves represent the mean ± sem of at least 3 independent experiments. Statistical analysis was performed using the two-way ANOVA and the square commas indicate the comparisons between the two curves. ** *p* < 0.01; ns non-significant.

Platelets have been shown to bind tumor cells shielding them from the cytotoxic activity of NK cells [25,26]. We thus investigated whether MM–platelet interactions could protect MM cells from NK cell-mediated cytotoxicity. To this end, we used the NK-sensitive MM cell line RPMI8226 since the MM1S cell line is resistant to NK cell-mediated cytotoxicity. SLe^a/x^-enriched and parental RPMI8226 cells were incubated with platelets as described before, washed and then co-cultured with expanded NK cells derived from the same platelet donor (platelet-autologous). The NK cells were mixed at different ratios with RPMI8226 cells and cell death was analyzed by flow cytometry. The RPMI8226 cells showed high sensitivity to cell death induced by NK cells, which was comparable between the SLe^a/x^-enriched and parental RPMI8226 (Figure S3). The presence of platelets decreased the response to NK cell-mediated cytotoxicity in the SLe^a/x^-enriched cells but not in the parental RPMI8226 cells (Figure 4A,B), suggesting that the physical interaction between platelets and MM is required to protect MM cells from the cytotoxic action of NK cells.

### 3.5. CD41/61 and SLe^a/x^ Double Positive Cells Are Present in Plasma Cells from MM Patients and Accumulate in Symptomatic Disease and at Relapse

**Figure 5:**
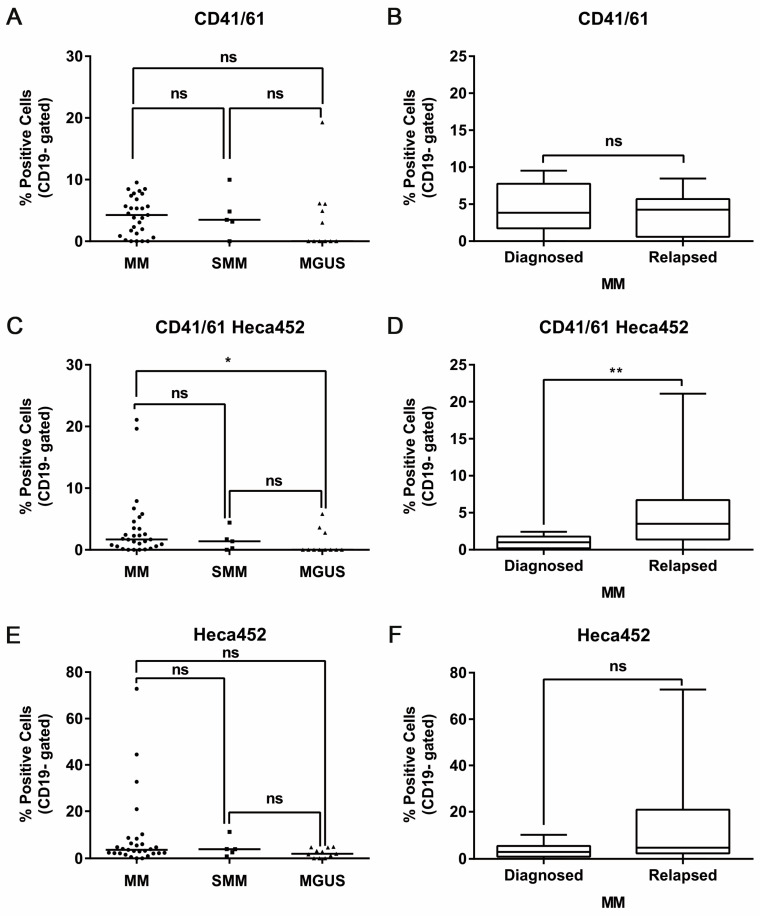
Platelets bind SLe^a/x^-enriched primary MM cells in BM samples of MM patients with significant enrichment in the symptomatic disease stage and at relapse. BM specimens were collected from MM patients in citrate, lysed in ammonium chloride-based buffer and stained for flow cytometry. Malignant plasma cells were defined as CD38+, CD138+, CD2-, CD14- and CD19- cells. Distribution of the CD41/61- (**A**), the CD41/61-HECA452 double positive (**C**) and the HECA452-positive (**E**) population at different disease stages. Horizontal lines represent the median. Box and whiskers showing the distribution of the CD41/61- (**B**), the CD41/61-HECA452 double positive (**D**) and the HECA452-positive (**F**) population in diagnosed versus relapsed MM patients. Statistical analysis was performed using either the two-way ANOVA and Sidak’s multiple comparison tests (**A**,**C**,**E**) or the non-parametric Mann-Whitney test (**B**,**D**,**F**). ** *p* < 0.01; * *p* < 0.05; ns non-significant.

We next asked whether the interactions between platelets and MM cells occurred also in vivo in BM aspirates from MM patients and therefore could play a role in the clinical course of the disease. First, 45 BM aspirates from 29 active myeloma patients, 5 SMM and 11 MGUS were analyzed. Tumor plasma cells were identified using a combination of markers including CD38, CD138, CD45, CD2, CD14, CD19 and CD56. The CD41/61 and the HECA452 antibodies were used to monitor the interactions between platelets and SLe^a/x^-enriched MM plasma cells, respectively. Through this approach, we could identify four different plasma cell populations: the single-positive HECA452 and CD41/61 cells, the double-positive CD41/61-HECA452 and the double-negative cells. The HECA452-positive, CD41/61-positive and double-positive cells were detected at all stages of the disease, suggesting that MM–platelet interactions occur from the asymptomatic to the symptomatic phase of the disease (Figure 5A,C,E). There was a significant enrichment of the CD41/61-HECA452 double-positive population in symptomatic MM compared to MGUS, indicating that this population increases during the progression of the disease (Figure 5C). Indeed, when we compared the distribution of the double positive CD41/61-HECA452 cells in samples from MM patients at diagnosis or relapse, we found a significant accumulation of this population at relapse (Figure 5D), suggesting that these cells could be selected by therapy. The same trend was observed in the HECA452 single positive cells, although it did not reach statistical significance (Figure 5E,F). The CD41/61 population was found only in primary samples and not in cell lines, indicating that in vivo myeloma cells may express additional ligands for platelet binding. There was no difference in the distribution of the CD41/61 single positive population between different disease stages or between MM patients (Figure 5A,B).

## 4. Discussion

Survival in the bloodstream represents a constant challenge to the malignant cells, as they are continuously subjected to adverse microenvironmental factors such as blood shear stress and cells of the immune system [27,28]. This is particularly relevant to MM, as malignant plasma cells are extremely dependent on the BM microenvironment for their survival. In this regard, we have previously identified a MM subpopulation that is enriched in SLe^a/x^, enabling MM cells to bind E-selectin, facilitating BM homing and retention [21,22]. In the present study, we have further investigated the biological features of these cells and uncovered a previously unappreciated high affinity for P-selectin. Indeed, Sle^a/x^-enriched MM cells show a strong adhesion to P-selectin compared to the parental cell lines, both under static as well as dynamic conditions. P-selectin is an important mediator of the interactions between the microenvironment and the MM cells [23]. In particular, it has been shown that monoclonal antibodies and small glycomimetic molecules (GMI-1070) that block the interactions between the MM cells and P-selectin reverse the microenvironment-induced in vitro and in vivo resistance to bortezomib, identifying P-selectin as a possible new therapeutic target in MM [23,29]. It is thus plausible that the strong binding to P-selectin observed in SLe^a/x^-enriched MM cells may cooperate with E-selectin to induce the aggressive phenotype and resistance to bortezomib exhibited by these cells in vivo [21,22].

An important role of platelets in MM progression has been proposed by Takagi et al., who showed that the activation status of platelets in the PB of MM patients correlates with the stage of the disease [16]. Of note, the MM cell lines were able to activate the platelets, which, in turn, induced an increase in MM cell proliferation in vitro and contributed to tumor engraftment in vivo [16]. We have now expanded these observations by showing for the first time a direct interaction between the MM cells and platelets. Binding to the platelets was demonstrated by flow cytometry and ImageStream analysis, which unambiguously showed CD41/61-positive platelets surrounding CD138-positive MM cells. Importantly, only the SLe^a/x^-enriched MM cells were able to directly bind the platelets, suggesting the requirement of this tetrasaccharide. Moreover, platelet binding was largely dependent on P-selectin expressed on platelets, as it could be blocked by an anti-P-selectin blocking antibody, correlating platelet interactions with the strong affinity for P-selectin exhibited by SLe^a/x^-enriched MM cells. These data strongly suggest that the binding between the platelets and SLe^a/x^-enriched MM cells may be mediated by a P-selectin ligand. An obvious candidate would be PSGL1, a putative mucin-like P-selectin ligand expressed at high levels in the MM cells [23]. Analysis of PSGL1 expression revealed no difference between the SLe^a/x^ and parental lines. However, our data do not completely rule out PSGL1 as the mediator of platelet binding. A suggestive hypothesis is that the tetrasaccharide SLe^a/x^ may decorate only a fraction of PSGL1, which would then be present in a large amount in the SLe^a/x^-enriched MM cells. In this scenario, the analysis of the expression levels of PSGL1 alone would not be enough to extrapolate its function, similar to what has been observed for other selectin ligands [30,31]. Indeed, it has been recently shown that PSGL1 is the main carrier of the Siglec-7 ligands, important molecules involved in MM immune evasion [32]. A detailed biochemical analysis of PSGL1 in the SLe^a/x^-enriched cells is thus warranted to conclusively establish its role in platelet binding.

Platelets have been shown to facilitate metastasis by forming a “cloak” on the surface of the malignant cells, protecting them from NK cell-mediated cytotoxicity [12,15,25,26]. Indeed, we observed that in sensitive MM cells, platelets are capable of decreasing NK cell-mediated cytotoxicity. Only the SLe^a/x^-enriched MM cells were protected from cell death induced by NK cells, emphasizing the central role of SLe^a/x^ and the direct binding to platelets in this process. Moreover, since SLe^a/x^-enriched MM cells exhibit a greater ability to metastasize due to the expression of selectin ligands on their surface [21,22], the presence of SLe^a/x^ could thus represent a unique advantage for the MM cells, combining a high metastatic potential with the ability to evade the immune system by platelet cloaking.

We have previously shown that SLe^a/x^-enriched cells are present in primary BM aspirates from MM patients [21]. These cells are characterized by an immature immune phenotype and tend to accumulate in patients at relapse. Here, we show that the SLe^a/x^-enriched primary MM cells also bind platelets in BM samples from MM patients. This cell population was observed in all disease stages analyzed, with significant enrichment in symptomatic MM compared to MGUS. The interactions between the platelets and SLe^a/x^-enriched primary MM cells were also significantly higher in BM samples from relapsed MM patients compared to those examined at diagnosis. These data strongly suggest that SLe^a/x^-enriched primary MM cells may be positively selected during the progression of the disease as well as by therapy. We also found a subpopulation of primary cells negative for SLe^a/x^ but positive for CD41/61, which was never observed in the cell lines analyzed in this study. Primary MM cells may harbor additional ligands for platelet binding that were not found in the cell lines analyzed [8,9,12]. We did not observe any correlation between this population and disease progression. However, the biological features of these cells may be important to fully understand the role of platelets in MM and require further investigation.

MM is characterized by the coexistence of multiple subclones, a feature known as intraclonal heterogeneity, which is present even at the MGUS stage [33]. Eventually, only a few of these subclones undergo progressive expansion that ultimately culminates in the establishment of a symptomatic disease [34]. Moreover, since genetic complexity can already be detected at the asymptomatic stages, factors other than genetic abnormalities may cooperate in selecting those subclones that will eventually lead to the transformation into MM [35,36]. The microenvironment plays an essential role in this process, as it represents a reservoir of pro-survival and proliferative signals that foster intraclonal heterogeneity and also provides the selective pressure that leads to the emergence of the fittest subclones. Platelets are a crucial component of the MM microenvironment as they support proliferation and engraftment in vivo [16]. Our studies emphasize the role of platelets in MM, as their direct engagement with MM cells depends on P-selectin, a molecule that has been shown to regulate the resistance of MM cells to chemotherapy [23]. Therefore, not only do platelets protect MM cells from NK cell-mediated cytotoxicity, but they may also be involved in cell adhesion-mediated drug resistance through the PSGL1/P-selectin axis, making them a valuable target for novel MM therapies.

## 5. Conclusions

In this study, we showed that the SLe^a/x^-enriched MM population exhibits a high affinity to P-selectin, which enables binding to platelets. Together with the strong binding to E-selectin as previously described [21], these biological features allow the MM population to become highly metastatic by enhancing their ability to escape the immune system and migrate to the BM niches. Importantly, these biological traits can be therapeutically targeted. Indeed, we showed that platelet–MM interactions could be disrupted by a P-selectin blocking antibody, even after the binding to platelets had been established. New therapeutic agents such as humanized monoclonal antibodies that block P-selectin or small glycomimetic molecules such as GMI-1070 are under development [23,29] and should be considered as possible therapeutic strategies for the management of MM patients.

## Data Availability

The data that support the findings of this study are available from the corresponding author upon reasonable request.

## Supplementary Materials

The following supporting information can be downloaded at: https://www.mdpi.com/article/10.3390/cancers15072154/s1, Figure S1: PSGL1 expression levels are comparable between SLe^a/x^-enriched and parental MM cells; Figure S2: The CD41/61-positive MM cells are also positive for the expression of SLe^a/x^; Figure S3: NK cell-mediated cytotoxicity in SLe^a/x^ versus parental MM cells in the absence of platelets; Table S1: Patients’ characteristics of MM cohort analyzed in this study.
