# Supplementary material for: Sialofucosylation Enables Platelet Binding to Myeloma Cells via P-Selectin and Suppresses NK Cell-Mediated Cytotoxicity
Source: Cancers (Basel). 2023 Apr 5;15(7):2154. doi: 10.3390/cancers15072154 (PMC10093642; doi:10.3390/cancers15072154)
Supplement: Supplementary file 1 [file cancers-15-02154-s001.zip › Supplementary Figures.pdf]

Supplementary Figure S1

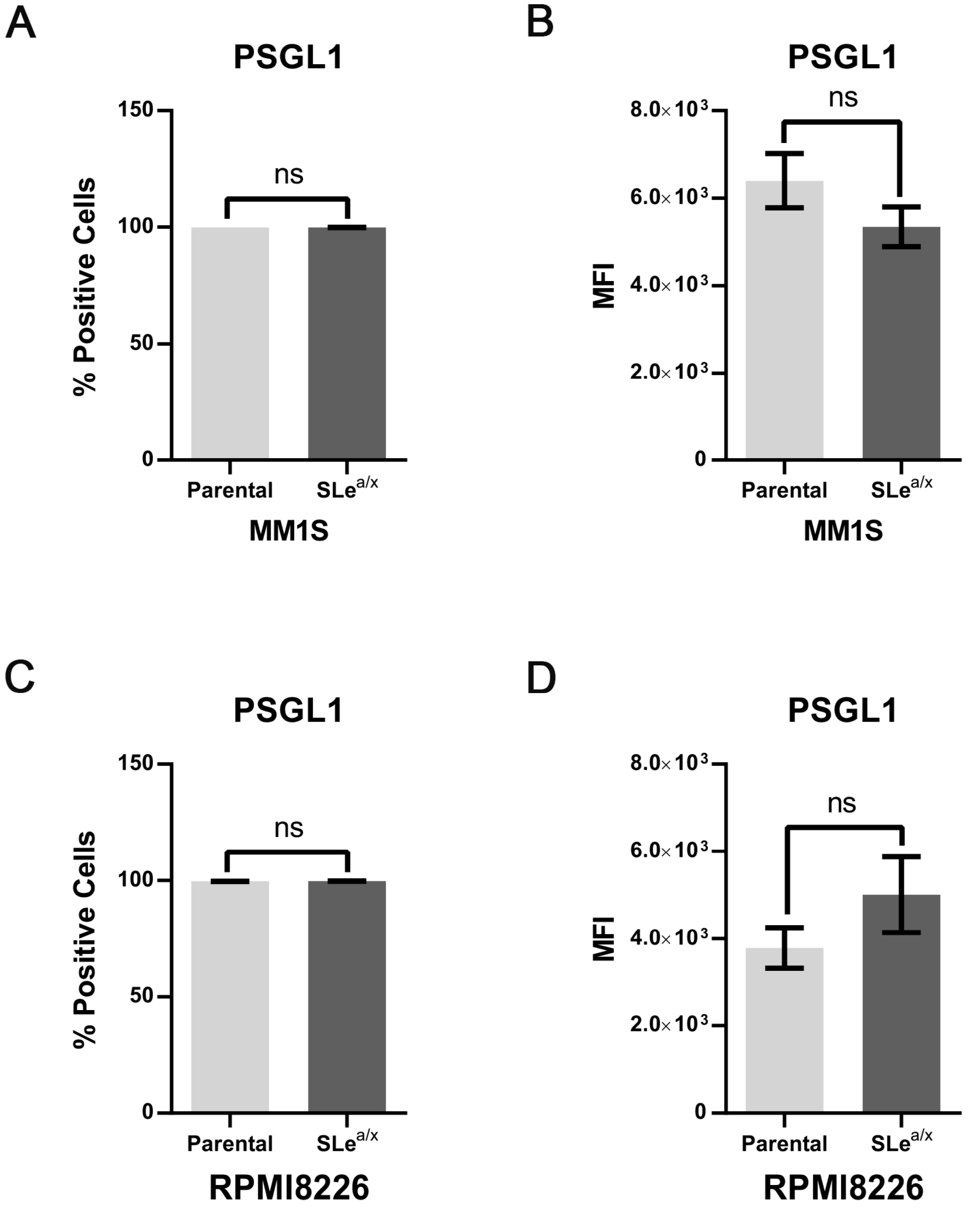

Supplementary Figure S1. PSGL1 expression levels are comparable between SLe$^{a/x}$ enriched and parental MM cells. MM1S (**A**,**B**) and RPMI8226 (**C**,**D**) SLe$^{a/x}$ enriched and parental cells were stained with the Heca452 and PSGL1 antibodies for 30 min, washed and analyzed by flow cytometry. Bars represent the mean ± sem of three independent experiments. Statistical analysis was performed using unpaired t test. ns non-significant.

Supplementary Figure S2

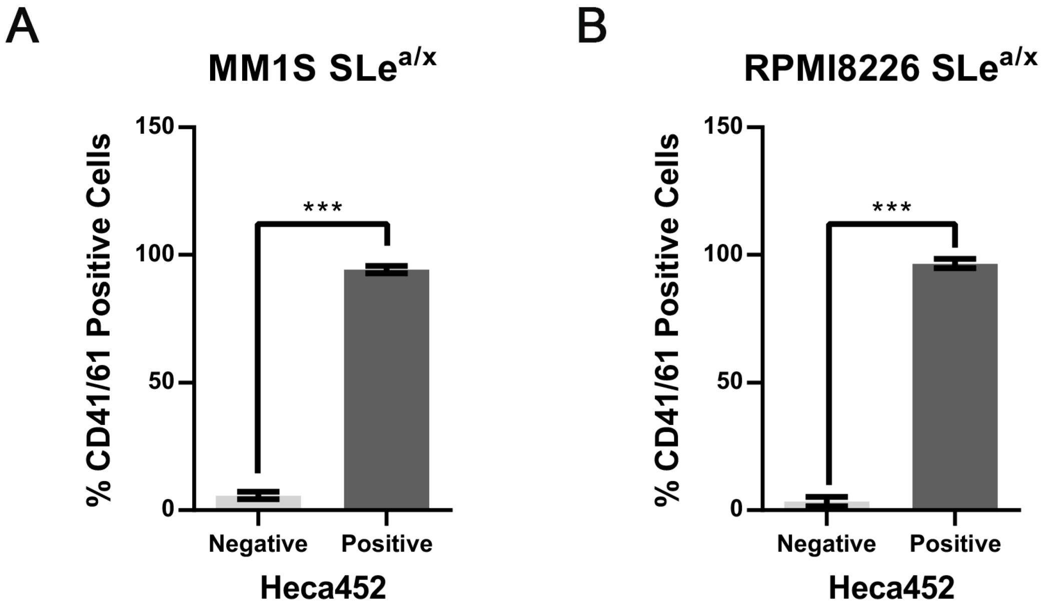

Supplementary Figure S2. The CD41/61-positive MM cells are also positive for the expression of SLe$^{a/x}$. MM cells were incubated with platelets and stained for flow cytometry as described in the main text. The percentage of CD41/61-positive cells was analyzed in the SLe$^{a/x}$ fraction of the enriched MM1S (**A**) and RPMI8226 (**B**) cells. Bars represent the mean ± sem of a least three independent experiments. Statistical analysis was performed using unpaired t test. *** $p < 0.001$.

Supplementary Figure S3

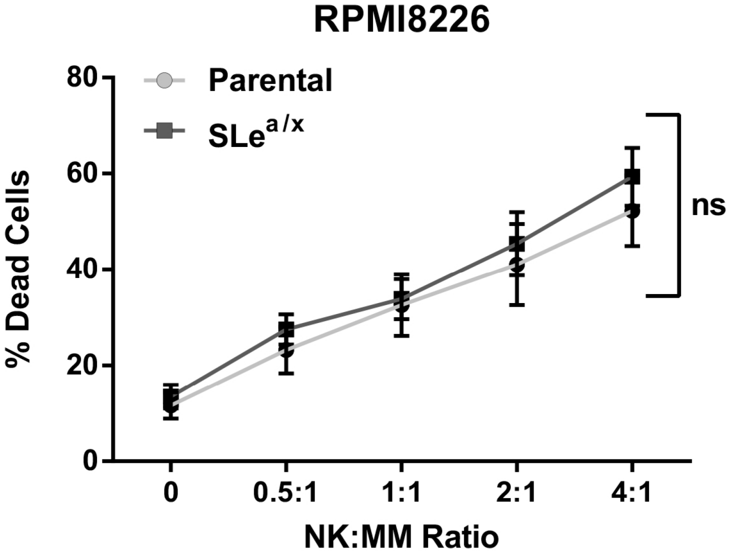

Supplementary Figure S3. NK cell-mediated cytotoxicity in SLe$^{a/x}$ versus parental MM cells in the absence of platelets. The SLe$^{a/x}$ enriched and parental RPMI8226 cells were treated as described in Figure 4 but without incubation with platelets. NK cell-mediated cytotoxicity was analyzed by flow cytometry using the Annexin V/7AAD assay. Points on the curves represent the mean ± sem of at least 3 independent experiments. Statistical analysis was performed using the two-way ANOVA and the square commas indicate the comparisons between the two curves. ns non-significant.
