# Supplementary material for: Sialofucosylation Enables Platelet Binding to Myeloma Cells via P-Selectin and Suppresses NK Cell-Mediated Cytotoxicity
Source: Cancers (Basel). 2023 Apr 5;15(7):2154. doi: 10.3390/cancers15072154 (PMC10093642; doi:10.3390/cancers15072154)
Supplement: Supplementary file 1 [file cancers-15-02154-s001.zip › Supplementary Table S1.pdf]

Number	disease	phase of disease	Current therapy	% pc
1	MM	Relapse	DRd	70
2	MM	Relapse	CTX	15
3	MM	Diagnosis	NA	58
4	MM	Diagnosis	Rd	54
5	SMM	NA	Naive	4
6	MM	Diagnosis	VRd + ASCT	3
7	MM	Relapse	DRd	73
8	MM	Relapse	Pd	46
9	SMM	Follow up	Naive	15
10	MM	Diagnosis	VMP	35
11	MGUS	NA	Naive	3
12	MGUS	Follow up	Naive	4
13	SMM	Follow up	Naive	16
14	MM	Diagnosis	Kd	12
15	MM	Diagnosis	KRd	18
16	MM	Follow up	Lenalidomide	2
17	MGUS	Follow up	Naive	3
18	MM	Diagnosis	VTD	26
19	MM	Relapse	Rd	15
20	MM	Relapse	Rd	7
21	MM	Relapse	DRd	10
22	SMM	Follow up	Naive	11
23	MM	Relapse	DRd	6
24	MM	Diagnosis	Naive	5
25	MGUS	Diagnosis	Naive	6
26	MGUS	Diagnosis	Naive	17
27	MM	Relapse	DRd	4
28	MM	Diagnosis	VMP	28
29	MGUS	Diagnosis	Naive	5
30	MGUS	Diagnosis	Naive	15
31	SMM	Diagnosis	Naive	12
32	MGUS	Follow up	Naive	3
33	MGUS	Follow up	Naive	4
34	MM	Diagnosis	VRd	63
35	MGUS	Diagnosis	Naive	9
36	MGUS	Diagnosis	Naive	6
37	MM	Follow up	Naive	10
38	MM	Diagnosis	Naive	37
39	MM	Relapse	Rd	3
40	MM	Relapse	Naive	31
41	MM	Diagnosis	DRd	26
42	MM	Relapse	EloPd	32
43	MM	Relapse	IsaPd	9
44	MM	Relapse	IsaPd	2
45	MM	Relapse	DRd	40

Supplementary Table S1. Patients' characteristics of MM cohort analyzed in this study. CTX: cyclophosphamide; Kd: carfilzomib, dexamethasone**;** Pd: pomalidomide, dexamethasone; Rd: lenalidomide, dexamethasone; DRd: daratumumab, lenalidomide, dexamethasone; EloPd: elotuzumab, pomalidomide, dexamethasone; IsaPd: isatuximab, pomalidomide, dexamethasone; KRd: lenalidomide, dexamethasone; VMP: bortezomib, melphalan, prednisolone; VRd: Bortezomib, lenalidomide, dexamethasone; VTd: bortezomib, thalidomide, dexamethasone.
